# Unusual acylation of chloramphenicol in *Lysobacter enzymogenes*, a biocontrol agent with intrinsic resistance to multiple antibiotics

**DOI:** 10.1186/s12896-017-0377-y

**Published:** 2017-07-04

**Authors:** Wei Zhang, Justin Huffman, Shengying Li, Yuemao Shen, Liangcheng Du

**Affiliations:** 1 Shandong Provincial Key Laboratory of Synthetic Biology, Key Laboratory of Biofuel, Chinese Academy of Sciences, Qingdao Institute of Bioenergy and Bioprocess Technology, 266101 Qingdao, China; 2 Department of Chemistry, University of Nebraska-Lincoln, Lincoln, NE 68588-0304 USA; 3 State Key Laboratory of Microbial Technology, School of Life Science, Shandong University, Jinan, 250100 China

**Keywords:** *Lysobacter*, Antibiotic resistance, Chloramphenicol, Acylation

## Abstract

**Background:**

The environmental gliding bacteria *Lysobacter* are emerging as a new group of biocontrol agents due to their prolific production of lytic enzymes and potent antibiotic natural products. These bacteria are intrinsically resistant to many antibiotics, but the mechanisms behind the antibiotic resistance have not been investigated.

**Results:**

Previously, we have used chloramphenicol acetyltransferase gene (*cat*) as a selection marker in genetic manipulation of natural product biosynthetic genes in *Lysobacter*, because chloramphenicol is one of the two common antibiotics that *Lysobacter* are susceptible to. Here, we found *L. enzymogenes*, the most studied species of this genus, could still grow in the presence of a low concentration of chloramphenicol. Three chloramphenicol derivatives (**1**–**3**) with an unusual acylation pattern were identified in a *cat*-containing mutant of *L. enzymogenes* and in the wild type. The compounds included chloramphenicol 3'-isobutyrate (**1**), a new compound chloramphenicol 1'-isobutyrate (**2**), and a rare chloramphenicol 3'-isovalerate (**3**). Furthermore, a mutation of a global regulator gene (*clp*) or a Gcn5-related *N*-acetyltransferase (GNAT) gene in *L. enzymogenes* led to nearly no growth in media containing chloramphenicol, whereas a complementation of *clp* restored the chloramphenicol acylation as well as antibiotic HSAF production in the *clp* mutant.

**Conclusions:**

The results indicated that *L. enzymogenes* contains a pool of unusual acyl donors for enzymatic modification of chloramphenicol that confers the resistance, which may involve the Clp-GNAT regulatory system. Because *Lysobacter* are ubiquitous inhabitants of soil and water, the finding may have important implications in understanding microbial competitions and bioactive natural product regulation.

**Electronic supplementary material:**

The online version of this article (doi:10.1186/s12896-017-0377-y) contains supplementary material, which is available to authorized users.

## Background

**Fig. 1:**
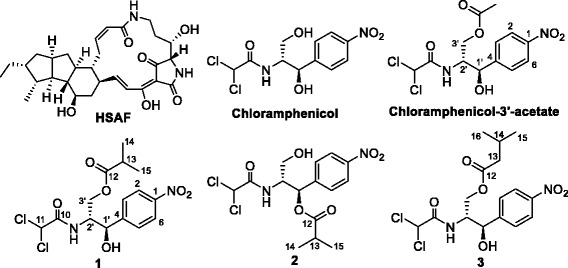
Chemical structure of the antifungal natural product HSAF and the unusual chloramphenicol derivatives (compounds **1**–**3**) produced in *Lysobacter enzymogenes* C3. **1**, chloramphenicol-3'-isobutyrate; **2**, chloramphenicol-1'-isobutyrate; **3**, chloramphenicol-3'-isovalerate. The structure of chloramphenicol and the usual product of chloramphenicol acyltransferase, chloramphenicol-3'-acetate, is also shown

*Lysobacter* is a genus of Gram-negative bacteria with high genomic G + C content ranging between 65 and 72%. As members of ecologically significant microbial communities ubiquitous in soil and aquatic environments, their agricultural relevance is becoming increasingly evident [1, 2]. Recent evidences also suggested that *Lysobacter* may occupy a wide range of ecological niches, including a broad range of ‘extreme’ environments [2–6]. Several *Lysobacter* species are prolific producers of lytic enzymes and bioactive natural products [1, 7]. These include multiple forms of *β*-1,3-glucanases and chitinases [8, 9] and potent antibiotics anti-MRSA cyclic peptides, such as lysobactin [10–12], tripropeptins [13, 14], and WAP-8294A [15–20]. Hybrid peptide-polyketides are also found in these bacteria, such as the cephem-type *β*-lactam cephabacins [21–23] and the antifungal compounds HSAF and analogs [24–33]. The latter group is particularly interesting because it has a distinct structure and unusual mode of action (Fig. 1). HSAF is the predominant antifungal compound produced by *L. enzymogenes* and has a novel mode of action against fungi. Thus, *L. enzymogenes* is considered a promising biocontrol agent against plant diseases caused by fungi, peronosporomycetes, nematodes, and bacteria, such as leaf spot of tall fescue caused by *Bipolaris sorokiniana* [34], bean rust caused by *Uromyces appendiculatus* [35] and *Fusarium* head blight of wheat [36].

As the most thoroughly characterized strain at both the molecular and biological levels [1, 7], *L. enzymogenes* is a genetically tractable species allowing for construction of gene knockouts, supporting its utility as a genetic model system for unraveling the molecular basis for mechanisms of microbial antagonism, biological control and natural products biosynthesis. However, *Lysobacter* species are naturally resistant to antibiotics commonly used in genetic selection in bacteria, such as kanamycin, ampicillin, streptomycin, tetracycline, and rifampin. This is a fairly unusual property for a group of ubiquitous environmental bacteria. Despite the potential as a new source of bioactive natural products and biocontrol agent, the majority of *Lysobacter* species remain unexplored, and the mechanism behind this very broad intrinsic antibiotic resistance is not known. An understanding of this mechanism is important because this knowledge could lead to new methods in genetic manipulation of this new emerging biocontrol agent. The finding could also have important implications in microbial ecology and agricultural application of the whole genus.

## Results

### Metabolites analysis of *L. enzymogenes* C3 and 5E4

In the process of investigating the antibiotic resistance in *Lysobacter*, we found that *L. enzymogenes* was able to survive in media containing chloramphenicol at 5 μg/mL or lower concentrations, and the OD_600_ can reach 1.1 after 3 days of incubation, which is also the normal cell density when incubated without any antibiotics. This is a surprising finding because chloramphenicol is one of the few antibiotic selection markers currently used in genetic manipulation in *L. enzymogenes*. Typically, chloramphenicol at 50 μg/mL is used to select against the wild type strain, while a genetic transformant of *L. enzymogenes* carrying the *cat* gene (encoding chloramphenicol acetyltransferase, CAT) [37], can grow normally under this concentration of chloramphenicol.

**Fig. 2:**
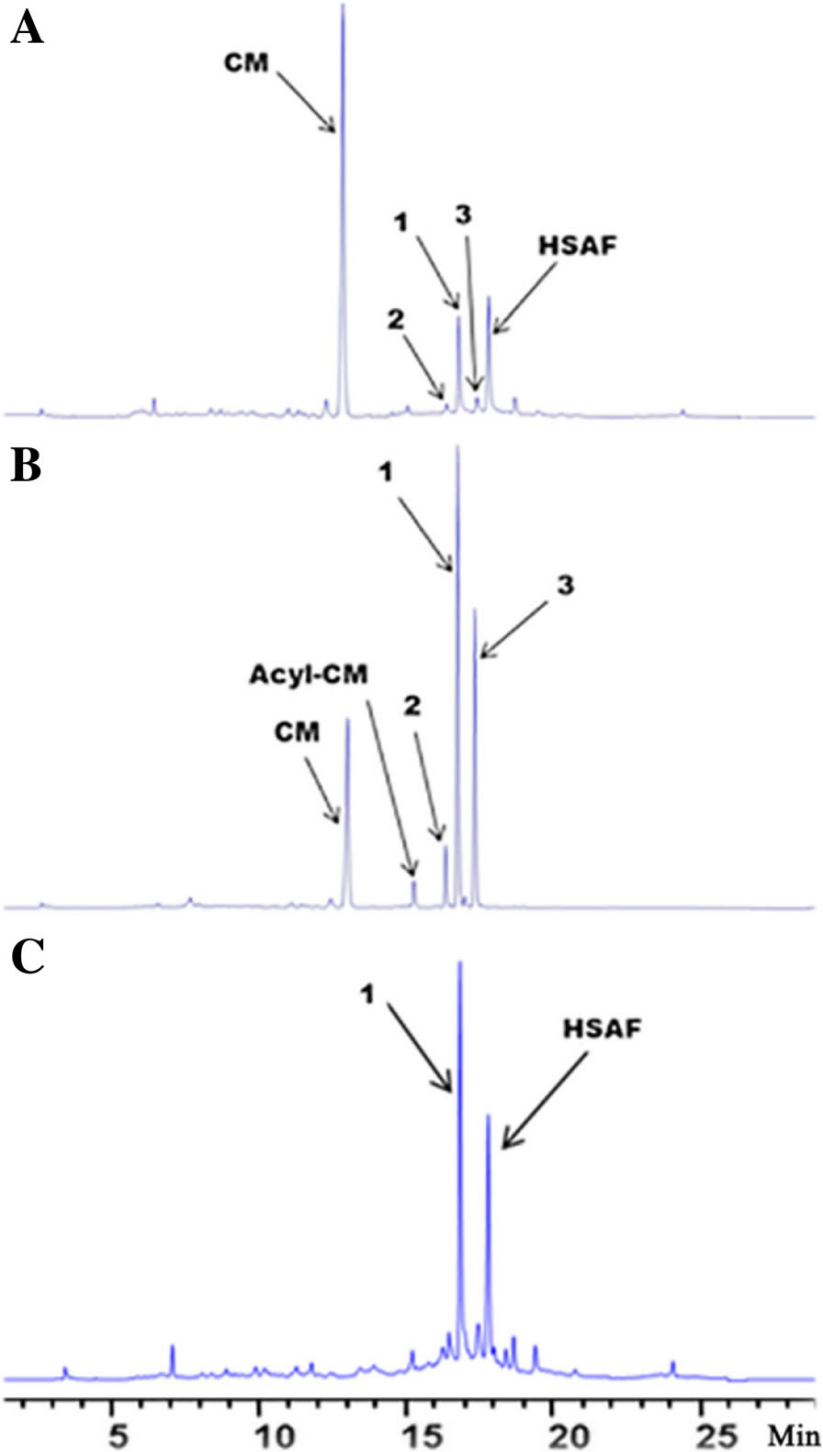
HPLC analysis of metabolites from the wild type *L. enzymogenes* C3 (**a**), from the transposon mutant 5E4 (**b**) and from *in trans* complemented strain (P2) of the *clp* mutant (5E4) (**c**). Strain C3 was grown in a medium containing 5 μg/mL chloramphenicol, strain 5E4 and strain P2 were in 50 μg/mL chloramphenicol. The peaks for chloramphenicol (CM), three acyl products of chloramphenicol (**1**, **2** and **3**), and HSAF are indicated with arrows. HR-LCMS also identified minor acyl derivatives (Acyl-CM) in the cultures. Note that acyl derivatives (indicated compound **1**, see Fig. 1 for structure) of chloramphenicol were also produced predominantly in the culture of strain P2﻿

To understand the chloramphenicol resistance, we searched for compounds derived from chloramphenicol in the wild type strain as well as in the mutant strain 5E4, which carried mini-Tn*5*-*lacZ*_1_-*cat* that was inserted into the *clp* gene encoding a global regulator belonging to the cAMP-receptor protein (CRP) family of transcriptional regulators [37]. On HPLC, several similar peaks appeared in the extracts from the wild type and strain 5E4 grown in a medium containing chloramphenicol (Fig. 2). One clear difference was that the wild type produced HSAF, but the *clp* mutant did not produce any detectable HSAF.

### Structure elucidation of chloramphenicol derivatives

Three main compounds (**1**, **2**, and **3**) were isolated from the cultures of 5E4. The ^1^H NMR spectra of compound **1** showed two doublets at *δ* 7.45 and 8.25, assigned to four aromatic protons (Additional file 1: Table S1), which indicates the presence of a 1,4-substituted benzene ring. The singlet at *δ* 6.22 (−CHCl_2_) and the complex resonance at *δ* 4.46 (m, H −2′) were identical to that of chloramphenicol. [38, 39] The DEPT experiment indicated the presence of one carbonyl group, suggesting compound **1** was a chloramphenicol ester. The acyl group was determined to be isobutyrate based on the molecular formula of compound **1** differing from that of chloramphenicol by C_4_H_7_O and the ^1^H NMR doublet at *δ* 1.17 integrated to six protons. The location of the isobutyrate group was determined at C-3′ hydroxyl based on the chemical shift of H-3′ at *δ* 3.5 in chloramphenicol has shifted to 4.30 and 4.42 ppm (Additional file 1: Table S1) [38, 39]. HRMS analysis gave a [M + Na]^+^ of 415.0440 (*calc*. 415.0440). Therefore, the structure of compound **1** was determined to be chloramphenicol-3′-isobutyrate (Fig. 1).

Compound **2** had a retention time on HPLC very close to compound **1** (Fig. 2) and showed a mass, [M + Na]^+^ of 415.0442 (*calc*. 415.0440), identical to **1**, and the only differences between these two structures are that the protons at *δ* 4.30 and 4.42 in **1** shifted to 3.50 and 3.66 ppm, and the proton at *δ* 5.10 in **1** shifted to 6.10 ppm (Additional file 1: Table S1), indicating the location of isobutyrate group at C-1′ hydroxyl in **2**. These ester linkages were further supported by the HMBC correlations between C-1′ and the ester carbonyl carbon in **2** and between C-3′ and the ester carbon in **1** (Additional file 1: Table S1). Therefore, compound **2** was determined to be chloramphenicol-1′-isobutyrate (Fig. 1), which is a new compound, and this acylation has not been recognized previously.

The NMR spectra of compound **3** were almost the same as **1**, except an additional methylene group. ^1^H-^1^H COSY showed the correlation between this methylene group and a methine group, which indicated that the isobutyrate group seen in **1** was replaced by an isovalerate group in **3** (Additional file 1: Table S1). HRMS analysis gave a [M + Na]^+^ of 429.0600 (*calc*. 429.0596). This compound was determined as chloramphenicol-3′-isovalerate (Fig. 1). This is the first NMR assignment for the structure, although this compound had been reported previously [38].

### Involvement of the global regulator CLP and the Gcn5-related *N*-acetyltransferase GNAT in the unusual acylations of chloramphenicol

The structural elucidation of these three compounds (**1**, **2**, and **3**) clearly showed that *L. enzymogenes* contains a pool of unusual acyl donors for modification of chloramphenicol. The acetyltransferase encoded by the *cat* gene in mini-Tn*5*-*lacZ*_1_-*cat* was originally from *E. coli* and typically catalyzes acetylation of 3′-hydroxyl of chloramphenicol to yield chloramphenicol-3′-acetate (Fig. 1). The unusual acylation at 1′- and 3′-hydroxyl with non-acetyl groups (isobutyrate and isovalerate) in mutant 5E4 suggested that *L. enzymogenes* C3 may have additional chloramphenicol detoxification mechanisms differing from that in *E. coli*. When the wild type strain C3 was cultured in a medium containing 5 μg/ml chloramphenicol, compounds **1**, **2** and **3** were also produced, albeit in a lower yield compared that from strain 5E4 (Fig. 2). LC-MS confirmed the chemical identity of the compounds. This suggested that the wild type may possess unusual acylation activity for chloramphenicol, presumably an intrinsic acyltransferase. In strain 5E4, the presence of the *cat* gene in the transposon enabled the cells to grow well even in a medium containing a high concentration (50 μg/mL) of chloramphenicol, which leads to a higher amount of **1**, **2** and **3** than the wild type. It is likely that the unusual acylation of chloramphenicol observed in strain 5E4 could be also partly due to this intrinsic acyltransferase activity in strain C3.

To further investigate this intrinsic acyltransferase activity, we tested several mutants of strain C3 generated by homologous recombination [37] without *cat* as the selectable marker. Mutant DC211 contained a 445-bp deletion within the global regulator gene *clp*. While strain DC211 grew normally in media without chloramphenicol, it barely grew in media containing 5 μg/mL chloramphenicol even after 3–4 days of incubation. This indicated that the *clp*-deletion mutant failed to detoxify the antibiotic by acylation, which consequently suppressed its growth in the presence of chloramphenicol even at 5 μg/mL. The result is in contrast to that from the wild type and suggests that the *clp* gene might be involved in the regulation of the intrinsic chloramphenicol resistance.

In the genome of *L. enzymogenes* C3, *clp* gene is tightly linked to and co-transcribed with an acyltransferase gene that belongs to the Gcn5-related *N*-acetyltransferase (GNAT) superfamily [37, 40, 41]. We tested the double mutant DCA2422 that lacks both *clp* and GNAT genes. Again, when this strain was inoculated into a medium containing 5 μg/mL chloramphenicol, there was nearly no growth of cells. Next, we tested the single mutant DA that resulted from a disruption of the GNAT gene alone and observed the same phenomenon as the double mutant. Because *clp* and the GNAT gene are expressed on a single RNA transcript [37], disruption of any of the genes is expected to have a nonfunctional mutant for both *clp* and GNAT. The observed loss of chloramphenicol resistance in all of these mutants suggests that the pair of Clp-GNAT regulators is involved in chloramphenicol detoxification in *L. enzymogenes* C3.

Finally, we tested a *clp*-complemented mutant, P2, in which another copy of the *clp* gene was inserted into the genome of mutant 5E4 [37]. P2 grew normally in the presence of chloramphenicol and also produced HSAF and the acylated chloramphenicols (Fig. 2). This result supports the involvement of the regulator in the resistance to chloramphenicol and is consistent with the role of Clp in regulation of HSAF biosynthesis [42–44]. It also implies that the *cat* gene of the mini-transposon (mini-Tn*5*-*lacZ*_1_-*cat*) in strain 5E4 may also contribute to the unusual acylations of chloramphenicol in *L. enzymegenes* C3, because the GNAT gene in P2 is nonfunctional.

**Fig. 3:**
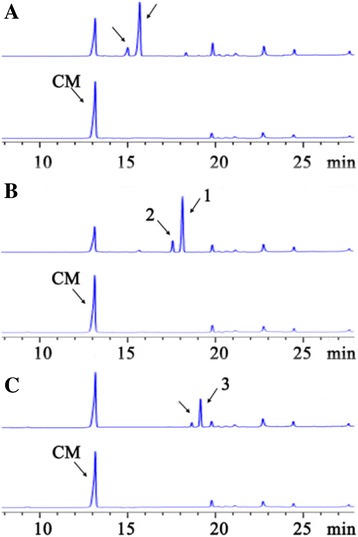
HPLC analysis of the reaction products of *E. coli* CAT with chloramphenicol and acetyl-CoA (**a**), isobutyryl-CoA (**b**), or isovaleryl-CoA (**c**) as substrate. In each pair of the HPLC traces, the top trace is from the reaction, and the bottom trace is from control (no CAT). Two acyl chloramphenicol products were produced in each of the reactions, as indicated by the arrows, with the first peak being the 1'-acyl chloramphenicol and the second the 3'-acyl chloramphenicol

To get more evidence, we tested if the *E. coli* CAT is able to catalyze the unusual acylation in vitro. We incubated purified CAT enzyme and acyl CoA (acetyl-CoA, isobutyryl-CoA or isovaleryl-CoA) in the presence of chloramphenicol. Because the purpose is to compare the yield of in vitro reactions with the yield of the 3-day *L. enzymogenes* culture, we kept the concentration of both substrates saturated, 280 μM chloramphenicol (*K*_m_ = 12 μM) and 250 μM acyl-CoA (*K*_m_ = 93 μM for acetyl-CoA) [45, 46]. The results showed that *E. coli* CAT gave a conversion rate of 59.9, 65.2, and 27.8%, respectively, when acetyl-CoA, isobutyryl-CoA, and isovaleryl-CoA was the substrate (Fig. 3). The results from isobutyryl-CoA and isovaleryl-CoA are surprising, but clearly show that CAT from *E. coli* is able to use alternative acyl-CoA as substrates. The in vitro results are supportive to the in vivo observations from various strains of *L. enzymogenes*. Apparently, the unusual acyl-CoA pool of isobutyryl-CoA and isovaleryl-CoA in *L. enzymogenes* is available to both the *E. coli* CAT encoded by the transposon and the native acyltransferase(s) of *L. enzymogenes*. The availability of the alternative acyl-CoA in the cellular pool most likely determines the unusual acylation of chloramphenicol as seen Fig. 2.

## Discussion

This is the first study on the mechanism for intrinsic antibiotic resistance in the genus of *Lysobacter*. The study identified three unusual acyl derivatives of chloramphenicol and showed the involvement of a global regulator and a GNAT in the chloramphenicol resistance. Chloramphenicol binds the 50S ribosomal subunit in prokaryotic cells and inhibits peptidyl transferase during protein biosynthesis [39]. Many bacteria inactivate chloramphenicol by chloramphenicol acetyltransferase, which catalyzes the acetylation of the primary hydroxyl using acetyl-CoA as substrate, yielding chloramphenicol-3′-acetate (Fig. 1). This action of CAT is the most common mechanism of chloramphenicol resistance in bacteria, such as *E. coli*, *Staphylococcus aureus*, *S. epidermidis*, and enteric bacteria [45]. In *L. enzymogenes* C3, we identified three compounds, chloramphenicol-3′-isobutyrate (**1**), a new compound chloramphenicol-1′-isobutyrate (**2**), and a rare chloramphenicol-3′-isovalerate (**3**). In the transposon mutant 5E4 containing the *cat* gene from *E. coli*, the major metabolite was chloramphenicol-3′-isobutyrate (**1**), rather than chloramphenicol-3′-acetate (Fig. 2). The in vitro activity assays showed that the *E. coli* CAT can use alternative acyl-CoA as substrate. Together, the results show that the difference in acylation products derives primarily from the availability of the acyl-CoA cellular pool and *L. enzymogenes* contains an acyltransferase(s) that can acylate chloramphenicol to confer the wild type with a low level chloramphenicol resistance. The results also imply that the available acyl-CoA pool in *L. enzymogenes* could be different from that in other bacteria.

The results also showed that the global regulator *clp* gene and the GNAT gene are involved in the observed chloramphenicol resistance. Additional experimentation is needed to establish the role of CLP in the chloramphenicol acylation. Whether this GNAT functions as regulator or could directly catalyze the unusual acylation reactions needs further biochemical studies.

## Conclusions

The study identified the structure of three unusually acylated derivatives of chloramphenicol and revealed a potential regulation through the global regulator CLP. Because *Lysobacter* are ubiquitous soil and water bacteria that produce several potent antibiotics and, in turn, are subjected to antibiotics produced by other microorganisms, the findings from this study provide important clues to how mechanisms of competition in *Lysobacter* are controlled. From an application perspective, the discovery of a regulatory mechanism for antibiotic biosynthesis could lead to new ways of metabolic engineering *Lysobacter* to improve the production of these compounds.

## Methods

### Chemicals, bacterial strains, and general procedures for DNA manipulation

Chemicals used in this study were purchased from Fisher Scientific or Sigma. *L. enzymogenes* C3 was used as the wild type strain. With the exception of DA, which is a GNAT disruption mutant of *L. enzymogenes* C3 developed in this study, all mutant strains were generated by Kobayashi et al. [37] and obtained from G. Yuen’s culture collection. Mutant strain 5E4 was generated using mini-Tn*5*-*lacZ*_1_-*cat*, which inserted between bases 222 and 223 from the start codon of the 690-bp *clp* gene. The *clp* gene deletion mutant DC211 contained a 445-bp deletion within the *clp* gene, beginning 182 bases from the start codon and 60 bases from the stop codon; the *clp*-GNAT double deletion mutant DCA2422 contained a 725-bp deletion between base 182 of the *clp* gene and base 211 of the 462-bp GNAT gene (there are 7 bases between *clp* and GNAT). Strain P2 was a *clp*-complementing strain of 5E4, which was generated by a chromosomal insertion of the *clp* gene into the *sctV* gene (part of a type III secretion system that is not associated with the *clp* gene or traits regulated by the gene). *Escherichia coli* DH5α strain was used as the host for general DNA propagations. *E. coli* S17-1 was used as the donor host strain for interspecies conjugation. All bacterial strains were grown in Luria-Bertani (LB) broth medium or 1/10-strength tryptic soy broth (1/10 TSB, Sigma). Genomic DNA of *L. enzymogenes* was prepared as previously described [37].

### Generation of DA mutant

A mutant strain of *L. enzymogenese* C3 disrupted at the GNAT gene was developed for this study. To construct the GNAT disruption vector, the PCR primers P1, 5′- TTA CTC GAG AGC TGC TGA GCC AGC TCG GCT-3′ (*Xho*I site underlined), and P2, 5′-AAC TGC AGG ACA CGT TGC TGG TGA CCT CG-3′ (*Pst*I site underlined), were designed to amplify an internal 302-bp region (78 base from the start codon and 82 base from the stop codon) within the 462-bp GNAT gene from the genomic DNA of *L. enzymogenes* C3. The amplified fragment was digested with *Xho*I/*Pst*I and cloned into the conjugation vector pJQ200SK [47] to yield pJQ200SK-AT. The pJQ200SK-AT construct was first transformed into *E. coli* S17-1 and then conjugally transferred from *E. coli* S17-1 into *L. enzymogenes* C3. Colonies that grew on plates containing 20 μg/mL gentamicin and 25 μg/mL kanamycin were considered as putative GNAT disruption mutants with the construct inserted into the GNAT site. To verify the putative mutant (strain DA), the primers P3, 5′-TGG CGG AAA CGG GAG-3′, and P4, 5′-ACC ATG ATT ACG CCA AGC-3′, were used to amplify a 434-bp fragment present in true mutants resulting from homologous recombination, but absent in the wild type or in mutants resulting from random insertion.

### Extraction and analysis of metabolites

To identify the new metabolites produced in cultures supplemented with chloramphenicol, *L. enzymogenes* C3 or its mutant was grown in 1/10 TSB for 1 day, and an aliquot of 2 mL was transferred to a 250 mL flask containing 50 mL of fresh 1/10 TSB. The cultures were incubated at 30 °C for 3 days with shaking at 200 rpm. To extract metabolites, culture fluid was collected by centrifugation, and the supernatant was extracted with an equal volume of ethyl acetate. The organic phase was dried with a rotavapor (Buchi, Rotavapor R-3) to afford the crude extract. The extract was dissolved in 1 mL methanol. A fraction (20 μL) of the extract was used for HPLC analysis on an Agilent 1200 with a reverse phase column (Agilent C18, 5 μ, 4.6 mm × 250 mm). HR-LCMS analysis of the chloramphenicol derivatives was performed on Dionex Ultimate 3000 with Bruker maxis Q-TOF using a Zorbax C18 column (2.1 × 100 mm, 1.8 μ). Water/0.1% formic acid (solvent A) and acetonitrile/0.1% formic acid (solvent B) were used as the mobile phases with a flow rate of 0.20 mL/min. The program was as follow: 5–20% B in A for the first 5 min, 20–80% B for the following 20 min, 80–100% B for 1 min, 100% B for 4 min, back to 5% B in 1 min and remaining for another 5 min. All the peaks were recorded at UV wavelength 318 nm. For HSAF analysis, the wild type C3 and mutant strains with various disruptions in the *clp*-GNAT pair of genes were grown in 1/10 TSB for 3–4 days. The supernatant was extracted by adding an equal volume of ethyl acetate. The ethyl acetate phase was collected and evaporated to dryness, and the residue was then dissolved in 1 mL methanol. A 20 μL aliquot of each extracts was analyzed by HPLC using the same Agilent system as described above. The mobile phases were water/0.1% trifluoroacetic acid (solvent A) and acetonitrile/0.1% TFA (solvent B), with a gradient of 5 to 40% mobile phase B in mobile phase A in the first 10 min, 40 to 80% B in A from 10 to 15 min, 80% B in A from 15 to 20 min, 80 to 100% B in A from 20 to 21 min, 100% B in A from 21 to 23 min, and 100 to 5% B in A from 23 to 25 min. The flow rate was 1.0 mL/min. The peaks were detected at 318 nm on a UV-visible detector (Agilent).

### Structural determination of chloramphenicol derivatives

To isolate the metabolites produced by mutant 5E4, one liter of fermentation broth was centrifuged and the cell mass discarded. The supernatant was extracted with ethyl acetate. The ethyl acetate extract was collected and evaporated to dryness in vacuum to afford 100 mg of extract. The extract (100 mg) was re-dissolved with MeOH and subjected to medium pressure liquid chromatography (10 g, RP-18, Waters, Ireland) and eluted sequentially with 100 mL of 30, 50, 70, and 100% MeOH in water. This yielded 4 fractions, Fr. S1-S4; Fr. S3 (48 mg) was subjected to HPLC purification, which gave 3 pure samples, **1** (25 mg), **2** (1.9 mg) and **3** (8 mg). To determine the structure of the compounds, HR-Q-TOF-MS data was acquired by using Bruker Q-TOF 6520 mass spectrometer. NMR spectra (^1^H, ^13^C, ^1^H-^1^H COSY and HMBC) were recorded on a Bruker DRX-500 spectrometer, at 500/125 MHz, respectively, in MeOD-*d*_6_, in *ppm* relative to Me_4_Si.

### In vitro chloramphenicol acyltransferase activity assay

Acyl-CoAs were purchased from Sigma, and *E. coli* CAT was purchased from Promega (part # E1051). The reaction contained 1.25 unit of CAT (one unit is defined as the amount of enzyme required to transfer 1 nmol of acetate to chloramphenicol in one minute at 37 °C), 250 μM acyl-CoA, 280 μM chloramphenicol, in 100 μl of 100 mM Tris buffer, pH 7.8. A reaction without CAT was served as control. The reaction was incubated at 37 °C for 2 h, and ethyl acetate (100 μL) was added to stop the reaction. The mixture was centrifuged (10,000 × g) for 10 min, and the ethyl acetate phase was collected and dried under a N_2_ flow. Methanol (100 μL) was added to dissolve the residues, and a fraction (20 μL) of the methanol solution was injected into HPLC for analysis (Agilent 1200 with a ZORBAX SB-C18 column, 4.6 mm × 150 mm, 5 μ). Acetonitrile (solvent B) and water (solvent A) were used as the mobile phases with a flow rate of 1.0 mL/min. The program was as follow: 10% B for 5 min, 10–90% B in A over 20 min, 90–100% B in A over 1 min, 100% B for 4 min, 100–10% B in A over 4 min, 10% B for 4 min. The detection wavelength was 220 nm. The peak integration area of the acylated chloramphenicol products on HPLC was used to calculate the conversion rate of each of the reactions with a different acyl-CoA substrate.

## Additional file

Additional file 1: Table S1. NMR spectroscopic data for compounds **1**, **2** and **3**.^*a*^ -^*a* 1^H-NMR and ^13^C-NMR spectra were obtained at 500 MHz and 125 MHz, respectively, and were recorded in CD_3_OD at room temperature. ^*b*^ Unless otherwise indicated, all proton signals integrate to ^1^H. (DOCX 14 kb)

## Abbreviations

*Cat*: Chloramphenicol acetyltransferase gene
CLP: cAMP receptor-like protein
CM: Chloramphenicol
COSY: Correlation spectroscopy
DEPT: Distortionless enhancement by polarization transfer
GNAT: Gcn5-related *N*-acetyltransferase
HMBC: Heteronuclear multiple bond correlation
HRMS: High resolution mass spectrometry
HSAF: Heat-stable antifungal factor

## References

[1] Christensen P, Cook FD (1978). Lysobacter, a new genus of non-fruiting, gliding bacteria with a high base ratio. Int J Syst Bacteriol.

[2] Sullivan RF, Holtman MA, Zylstra GJ, White JF, Kobayashi DY (2003). Taxonomic positioning of two biological control agents for plant diseases as Lysobacter enzymogenes based on phylogenetic analysis of 16S rDNA, fatty acid composition and phenotypic characteristics. J Appl Microbiol.

[3] Bae HS, Im WT, Lee ST (2005). Lysobacter concretionis sp. nov., isolated from anaerobic granules in an upflow anaerobic sludge blanket reactor. Int J Syst Evol Microbiol.

[4] Folman LB, Postma J, van Veen JA (2003). Characterisation of Lysobacter enzymogenes (Christensen and Cook 1978) strain 3.1T8, a powerful antagonist of fungal diseases of cucumber. Microbiol Res.

[5] Fukuda W, Kimura T, Araki S, Miyoshi Y, Atomi H, Imanaka T (2013). Lysobacter oligotrophicus sp. nov., isolated from an Antarctic freshwater lake in Antarctica. Int J Syst Evol Microbiol.

[6] Weon HY, Kim BY, Baek YK, Yoo SH, Kwon SW, Stackebrandt E, Go SJ (2006). Two novel species, Lysobacter daejeonensis sp. nov. and Lysobacter yangpyeongensis sp. nov., isolated from Korean greenhouse soils. Int J Syst Evol Microbiol.

[7] Xie Y, Wright S, Shen Y, Du L (2012). Bioactive natural products from Lysobacter. Nat Prod Rep.

[8] Palumbo JD, Yuen GY, Jochum CC, Tatum K, Kobayashi DY (2005). Mutagenesis of beta-1,3-Glucanase genes in Lysobacter enzymogenes Strain C3 results in reduced biological control activity toward Bipolaris leaf spot of tall fescue and pythium damping-off of sugar beet. Phytopathology.

[9] Zhang Z, Yuen GY, Sarath G, Penheiter AR (2001). Chitinases from the plant disease biocontrol agent, Stenotrophomonas maltophilia C3. Phytopathology.

[10] Bonner DP, O’Sullivan J, Tanaka SK, Clark JM, Whitney RR (1988). Lysobactin, a novel antibacterial agent produced by Lysobacter sp. II. Biological properties. J Antibiot (Tokyo).

[11] Hou J, Robbel L, Marahiel MA (2011). Identification and characterization of the lysobactin biosynthetic gene cluster reveals mechanistic insights into an unusual termination module architecture. Chem Biol.

[12] O’Sullivan J, McCullough JE, Tymiak AA, Kirsch DR, Trejo WH, Principe PA (1988). Lysobactin, a novel antibacterial agent produced by Lysobacter sp. I. Taxonomy, isolation and partial characterization. J Antibiot (Tokyo).

[13] Hashizume H, Hirosawa S, Sawa R, Muraoka Y, Ikeda D, Naganawa H, Igarashi M (2004). Tripropeptins, novel antimicrobial agents produced by Lysobacter sp. J Antibiot (Tokyo).

[14] Hashizume H, Igarashi M, Hattori S, Hori M, Hamada M, Takeuchi T (2001). Tripropeptins, novel antimicrobial agents produced by Lysobacter sp. I. Taxonomy, isolation and biological activities. J Antibiot (Tokyo).

[15] Harad KI, Suzuki M, Kato A, Fujii K, Oka H, Ito Y (2001). Separation of WAP-8294A components, a novel anti-methicillin-resistant staphylococcus aureus antibiotic, using high-speed counter-current chromatography. J Chromatogr A.

[16] Kato A, Hirata H, Ohashi Y, Fujii K, Mori K, Harada K (2011). A new anti-MRSA antibiotic complex, WAP-8294A II. Structure characterization of minor components by ESI LCMS and MS/MS. J Antibiot (Tokyo).

[17] Kato A, Nakaya S, Kokubo N, Aiba Y, Ohashi Y, Hirata H, Fujii K, Harada K (1998). A new anti-MRSA antibiotic complex, WAP-8294A. I. Taxonomy, isolation and biological activities. J Antibiot (Tokyo).

[18] Kato A, Nakaya S, Ohashi Y, Hirata H (1997). WAP-8294A(2), a novel anti-MRSA antibiotic produced by Lysobacter sp. J Am Chem Soc.

[19] Zhang W, Li Y, Qian G, Wang Y, Chen H, Li YZ, Liu F, Shen Y, Du L (2011). Identification and characterization of the anti-methicillin-resistant *Staphylococcus aureus* WAP-8294A2 biosynthetic gene cluster from *Lysobacter enzymogenes* OH11. Antimicrob Agents Chemother.

[20] Chen H, Olson AS, Su W, Dussault PH, Du L (2015). Fatty Acyl incorporation in the biosynthesis of WAP-8294A, a group of potent anti-MRSA Cyclic Lipodepsipeptides. RSC Adv.

[21] Harada S, Tsubotani S, Ono H, Okazaki H (1984). Cephabacins, new cephem antibiotics of bacterial origin. II. Isolation and characterization. J Antibiot (Tokyo).

[22] Ono H, Nozaki Y, Katayama N, Okazaki H (1984). Cephabacins, new cephem antibiotics of bacterial origin. I. Discovery and taxonomy of the producing organisms and fermentation. J Antibiot (Tokyo).

[23] Sohn YS, Nam DH, Ryu DD (2001). Biosynthetic pathway of cephabacins in Lysobacter lactamgenus: molecular and biochemical characterization of the upstream region of the gene clusters for engineering of novel antibiotics. Metab Eng.

[24] Li S, Du L, Yuen G, Harris SD (2006). Distinct ceramide synthases regulate polarized growth in the filamentous fungus *Aspergillus nidulans*. Mol Biol Cell.

[25] Lou L, Chen H, Cerny RL, Li Y, Shen Y, Du L (2012). Unusual activities of the thioesterase domain for the biosynthesis of the polycyclic tetramate macrolactam HSAF in *Lysobacter enzymogenes* C3. Biochemistry.

[26] Lou L, Qian G, Xie Y, Hang J, Chen H, Zaleta-Rivera K, Li Y, Shen Y, Dussault PH, Liu F (2011). Biosynthesis of HSAF, a tetramic acid-containing macrolactam from *Lysobacter enzymogenes*. J Am Chem Soc.

[27] Yu F, Zaleta-Rivera K, Zhu X, Huffman J, Millet JC, Harris SD, Yuen G, Li XC, Du L (2007). Structure and biosynthesis of heat-stable antifungal factor (HSAF), a broad-spectrum antimycotic with a novel mode of action. Antimicrob Agents Chemother.

[28] Ding YJ, Li YY, Li ZY, Zhang JL, Lu CH, Wang HX, Shen YM, Du LC (2016). Alteramide B is a microtubule antagonist of inhibiting Candida albicans. Bba-Gen Subjects.

[29] Ding YJ, Li ZY, Li YY, Lu CH, Wang HX, Shen YM, Du LC (2016). HSAF-induced antifungal effects in Candida albicans through ROS-mediated apoptosis. RSC Adv.

[30] Li Y, Chen H, Ding Y, Xie Y, Wang H, Cerny RL, Shen Y, Du L (2014). Iterative assembly of two separate polyketide chains by the same single-module bacterial polyketide synthase in the biosynthesis of HSAF. Angew Chem Int Ed Engl.

[31] Li YY, Huffman J, Li Y, Du LC, Shen YM (2012). 3-Hydroxylation of the polycyclic tetramate macrolactam in the biosynthesis of antifungal HSAF from Lysobacter enzymogenes C3. Med Chem Commun.

[32] Olson AS, Chen HT, Du LC, Dussault PH (2015). Synthesis of a 2,4,6,8,10-dodecapentanoic acid thioester as a substrate for biosynthesis of heat stable antifungal factor (HSAF). RSC Adv.

[33] Xu LX, Wu P, Wright SJ, Du LC, Wei XY (2015). Bioactive Polycyclic Tetramate Macrolactams from Lysobacter enzymogenes and their absolute configurations by Theoretical ECD Calculations. J Nat Prod.

[34] Zhang Z, Yuen GY (1999). Biological control of Bipolaris sorakiniana on tall fescue by Stenotrophomonas maltophilia strain C3. Phytopathology.

[35] Yuen GY, Steadman JR, Lindgren DT, Schaff D, Jochum C (2001). Bean rust biological control using bacterial agents. Crop Prot.

[36] Jochum CC, Osborne LE, Yuen GY (2006). Fusarium head blight biological control with Lysobacter enzymogenes. Biol Control.

[37] Kobayashi DY, Reedy RM, Palumbo JD, Zhou JM, Yuen GY (2005). A clp gene homologue belonging to the Crp gene family globally regulates lytic enzyme production, antimicrobial activity, and biological control activity expressed by *Lysobacter enzymogenes* strain C3. Appl Environ Microbiol.

[38] Argoudelis AD, Coats JH (1971). Microbial transformation of antibiotics. VI. Acylation of chloramphenicol by Streptomyces coelicolor. J Antibiot (Tokyo).

[39] El-Kersh TA, Plourde JR (1976). Biotransformation of antibiotics. I. Acylation of chloramphenicol by spores of Streptomyces griseus isolated from the Egyptian soil. J Antibiot (Tokyo).

[40] Poux AN, Marmorstein R (2003). Molecular basis for Gcn5/PCAF histone acetyltransferase selectivity for histone and nonhistone substrates. Biochemistry.

[41] Rojas JR, Trievel RC, Zhou JX, Mo Y, Li XM, Berger SL, Allis CD, Marmorstein R (1999). Structure of Tetrahymena GCN5 bound to coenzyme A and a histone H3 peptide. Nature.

[42] Han Y, Wang Y, Tombosa S, Wright S, Huffman J, Yuen G, Qian GL, Liu FQ, Shen YM, Du LC (2015). Identification of a small molecule signaling factor that regulates the biosynthesis of the antifungal polycyclic tetramate macrolactam HSAF in Lysobacter enzymogenes. Appl Microbiol Biot.

[43] Wang YS, Zhao YX, Zhang J, Zhao YY, Shen Y, Su ZH, Xu GG, Du LC, Huffman JM, Venturi V (2014). Transcriptomic analysis reveals new regulatory roles of Clp signaling in secondary metabolite biosynthesis and surface motility in Lysobacter enzymogenes OH11. Appl Microbiol Biot.

[44] Xu G, Shi X, Wang R, Xu H, Du L, Chou SH, Liu H, Liu Y, Qian G, and Liu F. Insights into the distinct cooperation between the transcription factor Clp and LeDSF signaling in the regulation of antifungal factors in Lysobacter enzymogenes OH11. *Biol Control.* 2017, in press.

[45] Day PJ, Shaw WV, Gibbs MR, Leslie AGW (1992). Acetyl Coenzyme-a Binding by Chloramphenicol Acetyltransferase - Long-Range electrostatic determinants of coenzyme-a recognition. Biochemistry.

[46] Murray IA, Shaw WV (1997). O-acetyltransferases for chloramphenicol and other natural products. Antimicrob Agents Ch.

[47] Quandt J, Hynes MF (1993). Versatile suicide vectors which allow direct selection for gene replacement in gram-negative bacteria. Gene.

