# Supplementary material for: Unusual acylation of chloramphenicol in Lysobacter enzymogenes, a biocontrol agent with intrinsic resistance to multiple antibiotics
Source: BMC Biotechnol. 2017 Jul 4;17:59. doi: 10.1186/s12896-017-0377-y (PMC5496308; doi:10.1186/s12896-017-0377-y)
Supplement: Additional file 1: Table S1. — NMR spectroscopic data for compounds 1, 2 and 3.a -a 1H-NMR and 13C-NMR spectra were obtained at 500 MHz and 125 MHz, respectively, and were recorded in CD3OD at room temperature. b Unless otherwise indicated, all proton signals integrate to 1H. (DOCX 14 kb) [file 12896_2017_377_MOESM1_ESM.docx]

**Table S1.** The NMR spectroscopic data for compounds **1**, **2** and **3**.*^a^*

| No. | **1** | | **2** | | **3** | |
| --- | --- | --- | --- | --- | --- | --- |
|  | *δ*_H_ (mult., *J* in Hz)*^b^* | *δ*_C_ | *δ*_H_ (mult., *J* in Hz) | *δ*_C_ | *δ*_H_ (mult., *J* in Hz) | *δ*_C_ |
| 1 |  | 147.2s |  | 145.2s |  | 147.2s |
| 2,6 | 8.21 (d, 9.0) | 122.8d | 8.26 (d, 7.0) | 122.8d | 8.21 (d, 7.0) | 122.8d |
| 3,5 | 7.68 (d, 8.5) | 127.0d | 7.65 (d, 8.5) | 127.0d | 7.68 (d, 8.5) | 127.0d |
| 4 |  | 149.4s |  | 140.7s |  | 149.4s |
| 1’ | 5.10 (d, 2.0) | 70.5d | 6.10 (d, 2.0) | 74.0d | 5.10 (d, 2.0) | 70.5d |
| 2’ | 4.46 (m) | 54.4d | 4.39 (m) | 55.8d | 4.43 (m) | 54.4d |
| 3’ | 4.42 (m),  4.30 (dt, 10.5, 2.5) | 63.5t | 3.66 (dd, 6.0, 11.5),  3.50 (dd, 5.5, 11.0) | 60.8t | 4.43 (m),  4.30 (dt, 10.5, 2.5) | 63.5t |
| 10 |  | 165.3s |  | 165.3s |  | 165.3s |
| 11 | 6.22 (s) | 65.8d | 6.30 (s) | 65.8d | 6.22 (s) | 65.8d |
| 12 |  | 177.0s |  | 177.0s |  | 177.0s |
| 13 | 2.61 (sept, 7.0) | 33.7d | 2.72 (sept, 7.0) | 34.3d | 2.23 (d, 7.0, 2H) | 44.1t |
| 14 | 1.17 (d, 7.5, 3H) | 17.8q | 1.17 (d, 7.5, 3H) | 19.1q | 2.10 (m) | 25.8d |
| 15 | 1.17 (d, 7.5, 3H) | 17.9q | 1.17 (d, 7.5, 3H) | 19.1q | 1.17(d, 7.5, 3H) | 17.9q |
|  |  |  |  |  | 1.17(d, 7.5, 3H) | 17.8q |

*^a^*^1^H-NMR and ^13^C-NMR spectra were obtained at 500 MHz and 125 MHz, respectively, and were recorded in CD_3_OD at room temperature.

*^b^*Unless otherwise indicated, all proton signals integrate to ^1^H.
